# On the Evolutionary Origin of Eukaryotic DNA Methyltransferases and Dnmt2

**DOI:** 10.1371/journal.pone.0028104

**Published:** 2011-11-30

**Authors:** Tomasz P. Jurkowski, Albert Jeltsch

**Affiliations:** School of Engineering and Science, Jacobs University Bremen, Campus Ring Bremen, Germany; Deutsches Krebsforschungszentrum, Germany

## Abstract

The Dnmt2 enzymes show strong amino acid sequence similarity with eukaryotic and prokaryotic DNA-(cytosine C5)-methyltransferases. Yet, Dnmt2 enzymes from several species were shown to methylate tRNA-Asp and had been proposed that eukaryotic DNA methyltransferases evolved from a Dnmt2-like tRNA methyltransferase ancestor [Goll et al., 2006, Science, 311, 395-8]. It was the aim of this study to investigate if this hypothesis could be supported by evidence from sequence alignments. We present phylogenetic analyses based on sequence alignments of the methyltransferase catalytic domains of more than 2300 eukaryotic and prokaryotic DNA-(cytosine C5)-methyltransferases and analyzed the distribution of DNA methyltransferases in eukaryotic species. The Dnmt2 homologues were reliably identified by an additional conserved CFT motif next to motif IX. All DNA methyltransferases and Dnmt2 enzymes were clearly separated from other RNA-(cytosine-C5)-methyltransferases. Our sequence alignments and phylogenetic analyses indicate that the last universal eukaryotic ancestor contained at least one member of the Dnmt1, Dnmt2 and Dnmt3 families of enzymes and additional RNA methyltransferases. The similarity of Dnmt2 enzymes with DNA methyltransferases and absence of similarity with RNA methyltransferases combined with their strong RNA methylation activity suggest that the ancestor of Dnmt2 was a DNA methyltransferase and an early Dnmt2 enzyme changed its substrate preference to tRNA. There is no phylogenetic evidence that Dnmt2 was the precursor of eukaryotic Dnmts. Most likely, the eukaryotic Dnmt1 and Dnmt3 families of DNA methyltransferases had an independent origin in the prokaryotic DNA methyltransferase sequence space.

## Introduction

DNA of most eukaryotic species is methylated, containing the modified base 5-methylcytosine. This modification has a major role in the silencing of gene expression, among other important functions [1], [2]. Broadly, eukaryotic DNA methyltransferases can be classified into the Dnmt1 and Dnmt3 families with several subfamilies [3], [4], [5], [6]. In prokaryotes, DNA methylation is observed at the C5 position of cytosine (cytosine-C5 methylation), but also at the exocyclic amino groups of adenine (adenine-N6 methylation) and cytosine (cytosine-N4 methylation). Prokaryotic DNA methyltransferases are mostly members of one of the several thousands of restriction-modification (RM) systems, which are involved in the protection of bacteria against bacteriophage infection [7]. The amino acid sequences and 3D structures of prokaryotic cytosine-C5 MTases are very similar to the methyltransferase domains of the eukaryotic enzymes, because all DNA-(cytosine-C5)-methyltransferases share a common set of ten characteristic amino acid sequence blocks [7], [8], [9] and a common fold [9]. The prokaryotic adenine-N6 and cytosine-N4 MTases are very similar to each other, but only distantly connected to cytosine-C5 MTases [7], [10]. In general, the evolution of DNA methyltransferases in eukaryotes is dominated by gene duplications and diversification combined with lineage specific loss of certain enzymes [6]. On the other hand, the evolution of prokaryotic DNA MTases is driven by divergence of the recognition sequences and massive horizontal gene transfer [10], [11].

In eukaryotes, another enzyme closely related to DNA-(cytosine-C5)-MTases called Dnmt2 had been identified by its sequence similarity to bacterial DNA methyltransferases [12], [13]. It belongs to a large family of proteins conserved from *S. pombe* to human, which implies an important functional role of this enzyme [14]. The Dnmt2 proteins contain all the sequence motifs characteristic for DNA-(cytosine C5)-MTases and the Dnmt2 structure strongly resemble prokaryotic DNA MTases [14], [15], but in contrast to all other mammalian DNA MTases, Dnmt2 does not possess a large N-terminal regulatory domain. Despite of the amino acid sequence and structural similarity, Dnmt2 biochemically showed only very weak DNA methylation activity [16], [17], [18], [19], [20], [21], [22]. In a seminal paper, Goll et al. (2006) demonstrated that Dnmt2 has a strong methylation activity at C38 of tRNA^Asp^ in mice, *Drosophila melanogaster* and *Arabidopsis thaliana* [23]. Unfortunately, the exact biological role of the Dnmt2 mediated tRNA methylation is not yet know. The position next to the anticodon loop may suggest a role in the basic transcriptional process, but influence on tRNA folding, and stability are also possible and recently a role of Dnmt2 in stress related tRNA processing has been observed [24].

Because of the importance of its discoveries, the Goll et al. (2006) paper [23] has become very influential and highly cited. In the same publication, it was suggested that the eukaryotic Dnmt1 and Dnmt3 DNA methyltransferases have evolved from a Dnmt2-like RNA methyltransferase ancestor that changed its target specificity from RNA to DNA [23]. Since no data were presented to support this interesting proposal, we investigated here if it could be backed up by molecular phylogeny or functional data.

## Results

### Generation of the multiple sequence alignment for phylogenetic analysis

**Figure 1:**
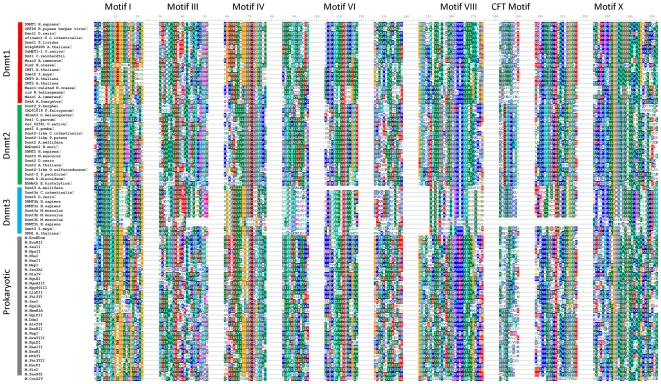
Multiple sequence alignment of the conserved amino acid sequence motifs from representative sequences of prokaryotic and eukaryotic DNA- and RNA-(cytosine-C5)-methyltransferases. The motif numbers are indicated on top of the sequence alignment. Note that the CFT motif is present only in Dnmt2.

**Figure 2:**
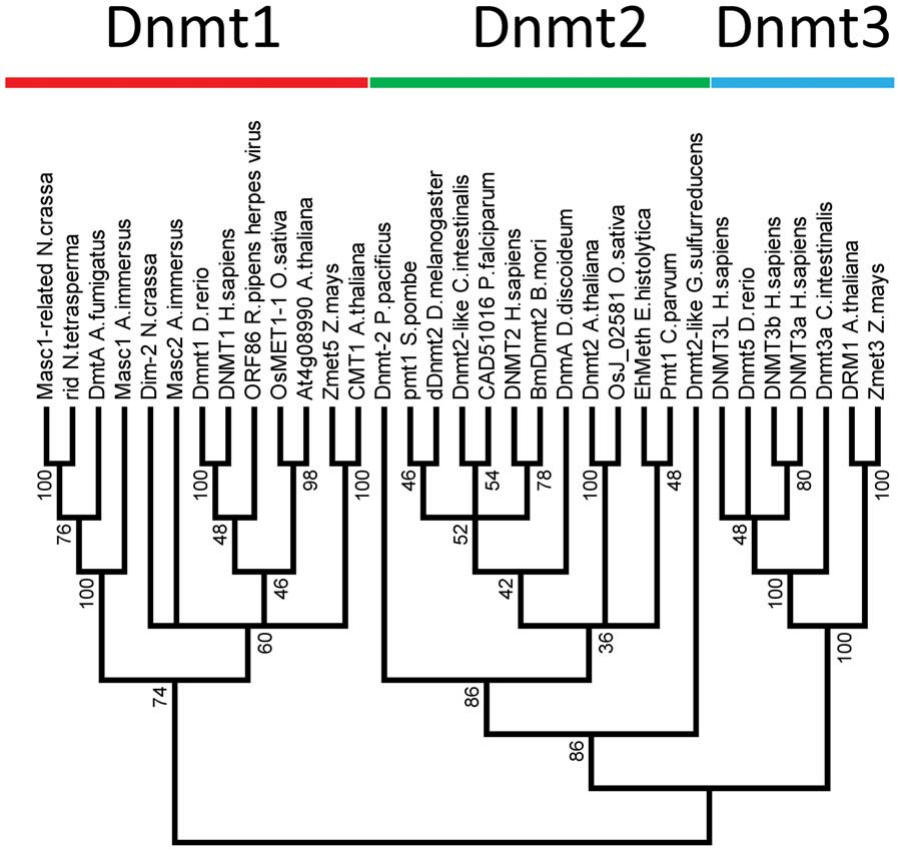
Consensus phylogenetic tree of the eukaryotic DNA-(cytosine C5)-MTases and Dnmt2 proteins constructed from 100 generated bootstrap trees. The bootstrap values of the branch points are indicated. Branch points with less than 30% incidence among the generated trees were collapsed representing that the phylogeny at this point cannot be reliably inferred.

**Figure 3:**
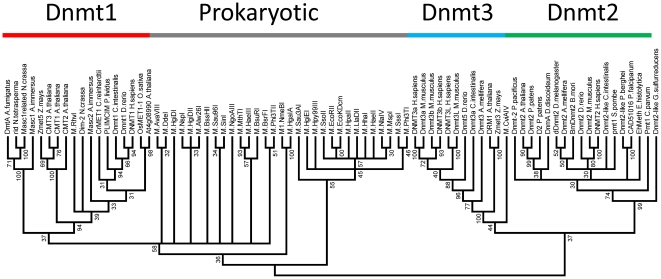
Consensus phylogenetic tree of prokaryotic and eukaryotic DNA-(cytosine C5)-MTases and Dnmt2 proteins constructed from 100 generated bootstrap trees. The bootstrap values of the branch points are indicated. Branch points with less than 30% incidence among the generated trees were collapsed representing that the phylogeny at this point cannot be reliably inferred.

We have prepared a multiple sequence alignment (MSA) of the conserved cytosine-C5 methyltransferase catalytic domains of more than 2300 prokaryotic and eukaryotic enzymes, comprising all sequences of eukaryotic DNA methyltransferases (Dnmt1 and Dnmt3 homologues, as well as plant and fungal DNA MTases), Dnmt2 proteins, bacterial and archeal DNA-(cytosine-C5)-MTases available in NCBI non-redundant and REBASE [25] databases. The Dnmt2 homologues can be reliably identified in the MSA, as they contain an additional conserved CFT motif next to motif IX. This complete alignment was used to calculate a guide tree, from which representatives of each major branch of the tree have been chosen for further analysis. The MSA of representative sequences was further evaluated and improved based on available crystallographic structures, fold recognition and secondary structure predictions (see the Methods section) (Fig 1). Using the refined MSA of the representative sequences, phylogenetic trees of prokaryotic and eukaryotic DNA-(cytosine-C5)-methyltransferases were generated. After removing the sequences of prokaryotic enzymes, we have also calculated a phylogenetic tree of eukaryotic DNA-(cytosine-C5)-methyltransferases only. Bootstrap analyses were conducted to evaluate the statistical significance of the branch points in both trees (Figs. 2 and 3).

### Evolution of eukaryotic DNA-(cytosine-C5)-methyltransferases and Dnmt2 from a eukaryotic perspective

The unrooted phylogenetic tree comprising eukaryotic DNA-(cytosine-C5)-methyltransferases and Dnmt2 homologues (Fig. 2) resembles a tree presented earlier [3]. It is roughly separated into three branches containing the Dnmt2, Dnmt3, Dnmt1 related enzymes, which all have highly significant bootstrap values (98, 100 and 74, respectively). The Dnmt1 clade comprises several clearly defined subgroups, the animal Dnmt1 enzymes, their plant Met1 counterparts, the CMT chromomethylases, as well as fungal Dim2, Masc2, RID, and Masc1 MTases. However, the mutual placement of these clades is not clear in some of the instances. The Dnmt2 clade contains many subgroups of Dnmt2 enzymes form the various eukaryotic lineages and one group of bacterial Dnmt2 related enzymes (represented by *Geobacter sulfurreducens*). The Dnmt3 clade is mainly split into animal Dnmt3 enzymes and plant DRM related enzymes.

The proposal that all eukaryotic DNA MTases arose from a Dnmt2-like enzyme implies that the root of this tree of eukaryotic MTases would lie within the Dnmt2 branch. However, there is no clear reason suggesting such placement of the root. In fact, the phylogenetic tree encompassing only eukaryotic DNA methyltransferases homologues and Dnmt2 neglects the presence of an enormous number of DNA methyltransferases present in prokaryotes. Consideration of these enzymes is crucial to deduce the phylogenetic history, as they are evolutionary connected to eukaryotic DNA MTases.

### Evolution of DNA-(cytosine-C5)-methyltransferases and Dnmt2 proteins from a global perspective

To investigate the phylogeny of eukaryotic DNA MTases and Dnmt2 proteins in the context of the prokaryotic enzymes, we have prepared an unrooted phylogenetic tree, which besides the eukaryotic enzymes mentioned above also contains representative sequences of prokaryotic enzymes (Fig. 3). The multiple sequence alignments of DNA MTases and Dnmt2 proteins with RNA-(cytosine-C5)-MTases indicated that RNA MTases are so different from DNA-(cytosine-C5)-MTases that it was not possible to reliably use them for rooting of the DNA MTase phylogenetic tree. This observation suggested that neither Dnmt2 nor any other known DNA methyltransferase shares a close evolutionary relationship with RNA methyltransferases. The lack of an appropriate outgroup also prevented us from rooting the tree, which would allow direct testing of the phylogenetic hypothesis raised above.

In the unrooted tree including the prokaryotic DNA-(cytosine C5)-MTases the main branches of the eukaryotic Dnmts were preserved. The prokaryotic MTases appeared in several branches with weak similarity between them. Most importantly, the Dnmt2, Dnmt3 and Dnmt1 MTases were separated by numerous branches of prokaryotic enzymes. However, the bootstrap values for this tree were less favorable for many of the branch points. We compared the individual guide trees used for the bootstrapping manually, to identify the reason for the weaker bootstrap values and realized that it was the flexible placement of some of the prokaryotic branches when changing from one tree to another, which changed the neighboring topology and caused the overall reduction in bootstrap values.

**Figure 4:**
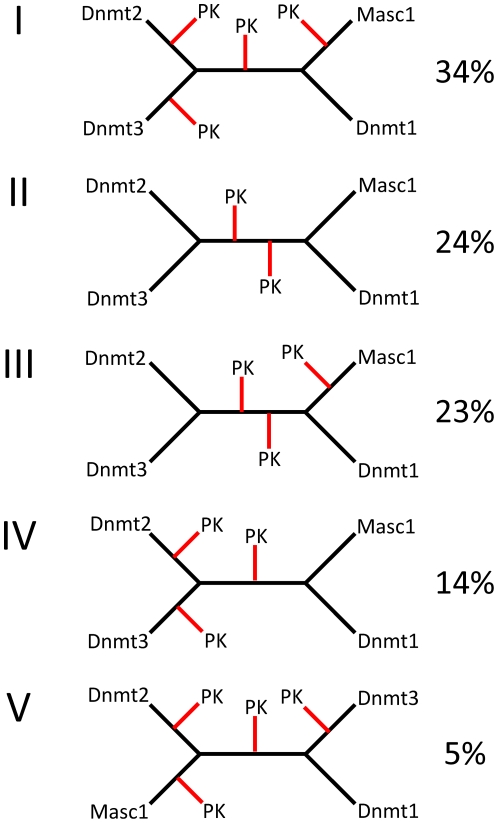
Compilation of different topologies 100 alternative the phylogenetic trees of prokaryotic and eukaryotic DNA-(cytosine C5)-MTases and Dnmt2 proteins generated during the bootstrapping analysis of the tree shown in Fig. 3.

It was the main goal of our work was to find out if the proposal that Dnmt2 was the precursor of Dnmt1 and Dnmt3 enzymes is supported by phylogenetic data. Such evolutionary scenario would result in a tree topology with Dnmt1, Dnmt2 and Dnmt3 clustering together separated from prokaryotic MTases. Therefore, we were mainly concerned with the overall topology of the tree. Bootstrap values are not the appropriate measure to determine the statistical significance of the general tree topology, because they evaluate the strength of each individual node. We, therefore, manually inspected 100 of the alternative trees used for bootstrapping and clustered them according to their topology (Fig. 4).

In 95% of the trees, the arrangement of the eukaryotic enzymes was identical to the eukaryotic tree with Dnmt2 and Dnmt3 enzymes clustering away from the Dnmt1 branch (topologies I-IV in Fig. 4) indicating that Dnmt2 and Dnmt3 are closer related to each other than to Dnmt1. However, in 63% of the trees different branches of prokaryotic enzymes were inserted between Dnmt2 and Dnmt3 (topologies I, II and V in Fig. 4). Within the Dnmt1 group, the placing of the Masc1 enzymes became less defined, because of the occasional insertion of different groups of prokaryotic enzyme between Dnmt1 and Masc1 (topologies I, II and V in Fig. 4). Most importantly in all of the 100 trees, the Dnmt1 and Dnmt3 enzymes were separated at least by some prokaryotic MTases. This analysis suggests that Dnmt1 and Dnmt3 are not monophyletic but they were derived from different prokaryotic DNA MTases. The same observation holds true for Dnmt1 and Dnmt2 indicating that Dnmt2 is not closely related to Dnmt1 as well.

### BLAST and CLANS analyses

**Figure 5:**
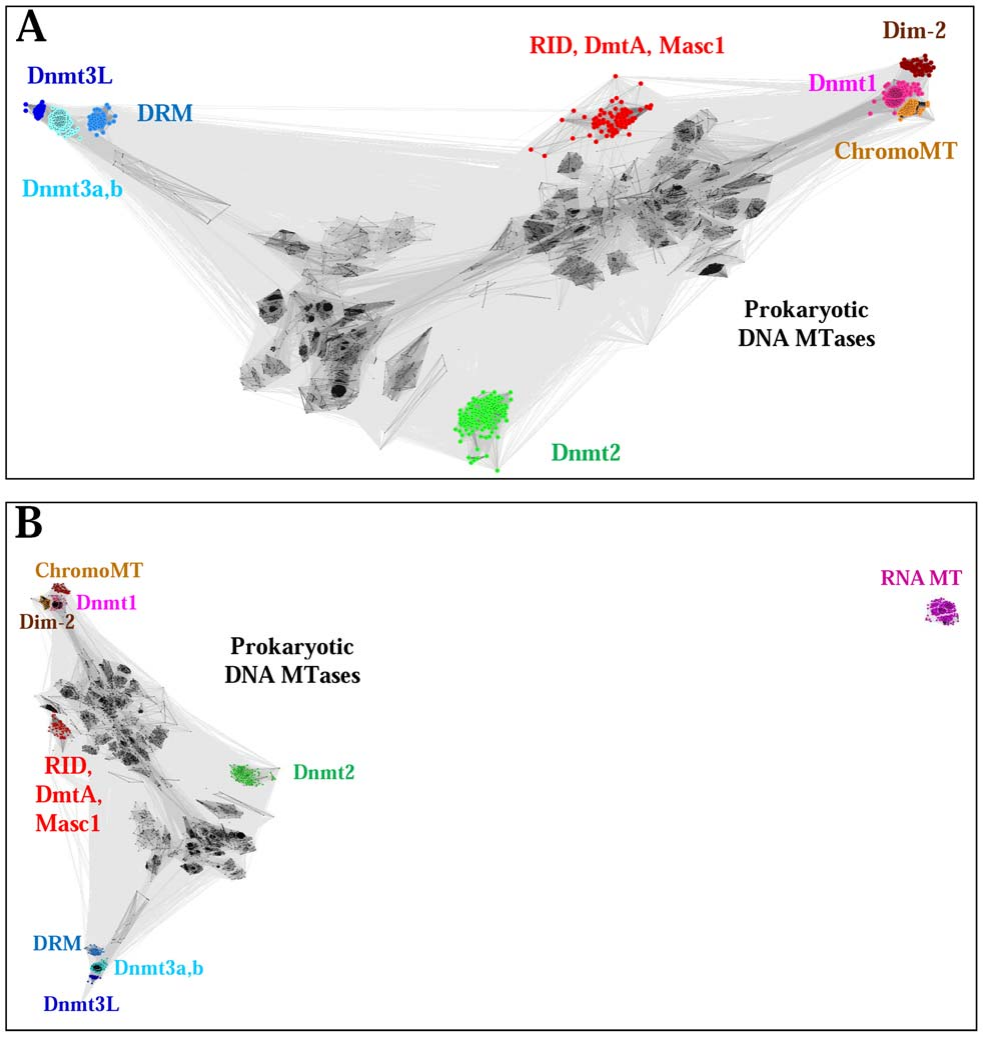
3D clustering of methyltransferase sequences based on pairwise BLAST similarity scores prepared using CLANS [26]. A) Clustering of 2935 sequences of prokaryotic and eukaryotic DNA-(cytosine C5)-MTases and Dnmt2 proteins. B) Clustering of the sequences of prokaryotic and eukaryotic DNA-(cytosine C5)-MTases and Dnmt2 proteins including sequences of RNA-(cytosine C5)-MTases.

To assess the sequence similarity of eukaryotic and prokaryotic DNA MTases and Dnmt2 enzymes with a second independent method, we performed a 3D clustering of 2935 sequences based on BLAST scores from pairwise alignments using CLANS [26] (Fig. 5A). The clustering identified all the major groups of eukaryotic Dnmts as described above. In addition the Dnmt1, Dnmt2 and Dnmt3 enzymes were clearly separated and all of them showed higher similarity to prokaryotic enzyme than to each other. Next, we also included RNA-(cytosine C5)-MTases into the clustering (Fig. 5B). The resulting distribution showed clearly that RNA MTases do not share close sequence similarity with prokaryotic or eukaryotic DNA MTases and Dnmt2 proteins. Most importantly, Dnmt2 enzymes share much higher similarity with DNA MTases than with RNA MTases. These results confirm all the conclusions from the multiple sequence alignments and tree building described above.

### Distribution of DNA methyltransferases and Dnmt2 proteins in eukaryotes

**Figure 6:**
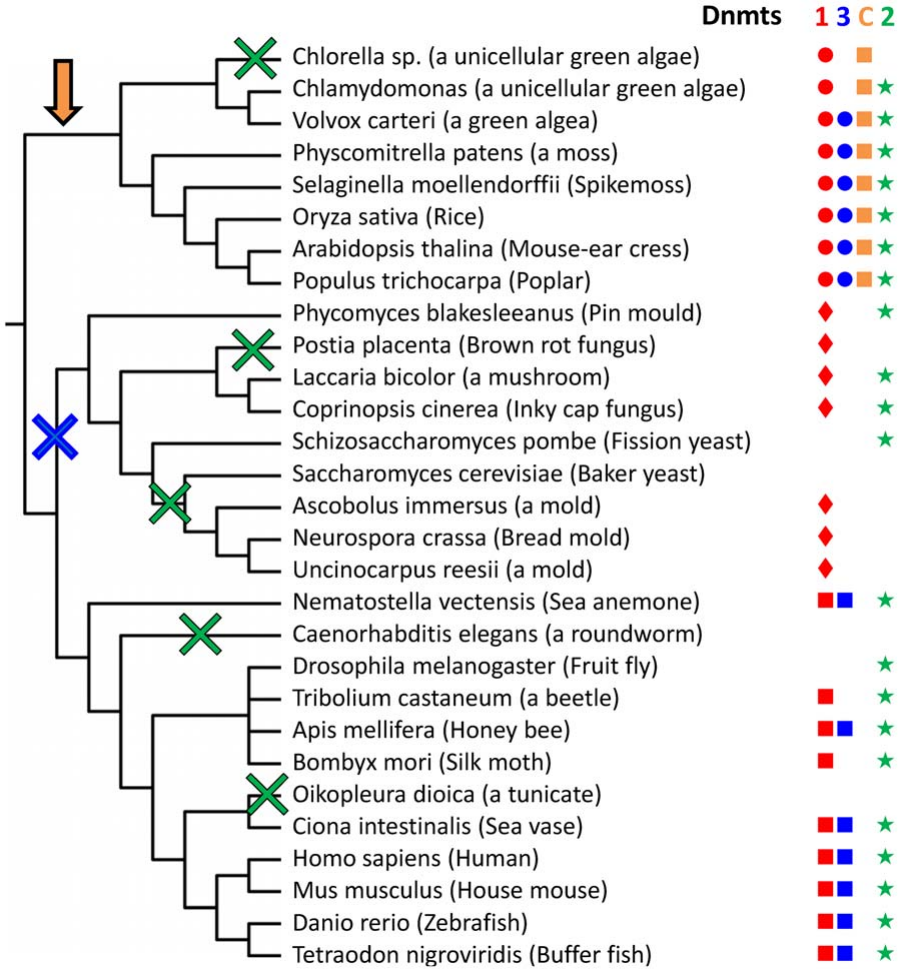
Distribution of the methyltransferases in different eukaryotic species. The tree was prepared using NCBI taxonomy and Interactive Tree Of Life. Dnmts were categorized into the Dnmt1 (1, colored red), Dnmt3 (3, colored blue), Chromomethylase (C, colored orange) and Dim2 families (D, colored green). The Dnmt1 enzymes were subdivided into animal, plant and fungi subgroups, Dnmt3 enzymes into the canonical Dnmt3 enzymes and the plant DRM enzymes. Lineages that underwent loss of Dnmt2 are indicated by green crosses. Dnmt1 orthologues are found in all the branches of eukaryotes. Chromomethylases are related to Dnmt1 enzyme appearing in the plant lineage only (indicated by the orange arrow). Dnmt3 enzymes apparently have been lost in the fungal lineage (indicated by a blue cross).

In order to better understand the evolution of eukaryotic DNA methyltransferases, we have analyzed the distribution of DNA methyltransferases in eukaryotic genomes. As illustrated in Fig. 6, the evolution of Dnmts in eukaryotes is characterized by gene duplications and loss of genes in some lineages. Most of the organisms analyzed here possess at least one Dnmt gene, with the exception of *Saccharomyces cerevisae*, *Caenorhabditis elegans* and *Oikopleura dioica*. These three organisms also do not contain any detectable level of DNA methylation [4], [5]. Loss of DNA-(cytosine C5)-methyltransferases generally can be explained by the mutagenic properties of 5-methylcytosine in DNA due to its deamination to thymidine which is more difficult to repair than uracil, the deamination product of unmethylated cytosine. In RNA deamination of 5-methylcytosine is not expected to be as critical as in DNA. However, the methylation of tRNAs requires resources, such that species adapted to rapid growth under good conditions, like *Saccharomyces cerevisiea*, may benefit from its omission.

Dnmt2 homologues are present in the majority of plant, fungi and animal species analyzed here and in *S. pombe* and *D. melanogaster* a Dnmt2 homologue is the only DNA MTase-like protein present in the genome. Such strong evolutionary conservation suggests a very important function of this enzyme. Dnmt2 enzymes were most likely lost from some fungal species, like in the phylum of Ascomycetes including *S. cerevisiae*; however a Dnmt2 homologue is present in the closely related species *S. pombe* (Pmt1) [27]. The nematode *C. elegans* also has lost the Dnmt2 gene, but it is present in the nematode *Pristionchus pacificus* [28]. Similarly, we could not identify any Dnmt2 enzyme in the genomes of *Chlorella sp.*, *Postia placenta* and *Oikopleura dioica*, but the presence of Dnmt2 in other related species suggest that this enzyme was specifically lost from these organisms.

Dnmt3 homologues are only present in the plant and animal kingdoms and were completely lost from fungi. All mammalian species contain a Dnmt3 homolog. In plants, the most primitive Dnmt3 homolog is the M.CviAIV methyltransferase found in *Chlorella* species [Gurnon et. al., unpublished observations cited in REBASE]. This is especially interesting, as this enzyme clusters together with eukaryotic Dnmt3 proteins in the phylogenetic tree, but it looks like a typical methyltransferase belonging to a RM system (not containing an N-terminal domain). The domain rearranged methyltransferases (DRM) are found only in plants - these enzymes most likely arose through Dnmt3 gene duplication and circular permutation [11], [29], [30].

Enzymes clustering together with Dnmt1 are present in plants, fungi and animals; however in each of the kingdoms they have their own sequence features. In plants, CMT chromomethylases and Met1 Dnmt1 orthologues are present; they most likely arose through gene duplication and specialization. Fungal genomes contain RIP-deficient methyltransferases and MASC1, Dim2 and MASC2 homologues as members of the Dnmt1 family, but biochemically none of them displayed a preference for hemimethylated DNA, suggesting that they may have an altered functional role. Also, in some insects, like *Bombyx mori* and *Tribolium castaneum,* only a Dnmt1 homolog is present, however the sequence specificities of these enzymes are not known yet. The wide distribution of Dnmts in all groups of eukaryotes strongly suggests that the last eukaryotic common ancestor (LECA) contained at least one Dnmt1, Dnmt2 and Dnmt3 gene.

## Discussion

Given the structural and mechanistic similarities of DNA and RNA cytosine-C5 specific methyltransferases [31], [32], [33], [34], an evolutionary relationship of these classes of enzymes can be assumed. It had been proposed that eukaryotic DNA methyltransferases evolved from a Dnmt2-like tRNA methyltransferase ancestor [23]. It was the aim of this study to investigate if this hypothesis could be supported by phylogenetic evidence derived from a sequence alignment of more than 2300 unique DNA MTases and Dnmt2 enzymes or if alternative models could be proposed.

### Did Dnmt2 derive from an RNA or DNA MTase?

**Figure 7:**
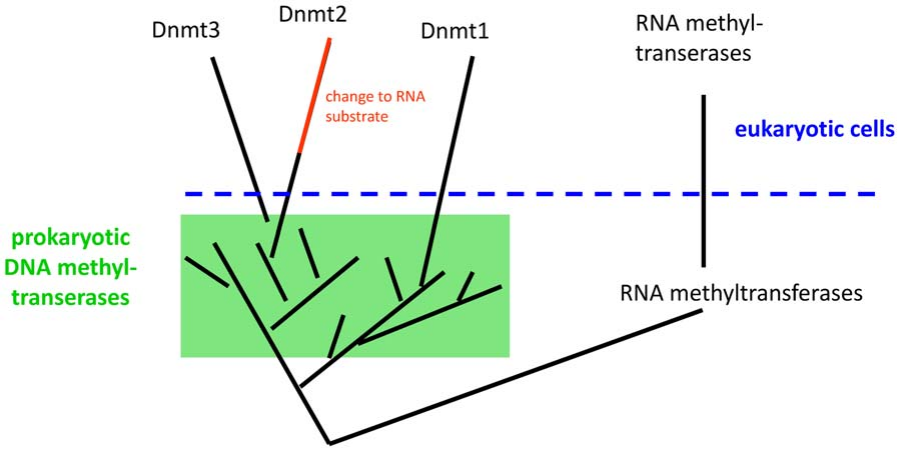
Consensus model of the phylogeny of DNA methyltransferases and Dnmt2 indicating that Dnmt1 and Dnmt2/3 enzymes have an independent origin in the prokaryotic DNA methyltransferases.

In the light of the general model that an RNA world preceded the current DNA world [35], [36], one might speculate that DNA methyltransferases were derived from RNA methyltransferases. However, if such transition happened at all, it must have occurred long before the development of eukaryotic cells, because the wide distribution of the Dnmt1, Dnmt2 and Dnmt3 enzyme families in eukaryotes clearly indicates that all these enzymes were present already in the last eukaryotic common ancestor (LECA) (Fig. 6). In general, Dnmt2 enzymes are not found in bacteria indicating that they were introduced in LECA. Dnmt2 clusters with eukaryotic and prokaryotic DNA MTases but not with RNA MTases. Therefore, the evolutionary precursor of Dnmt2 most likely was a prokaryotic DNA methyltransferase and not an RNA methyltransferase (Fig. 7). In LECA, Dnmt2 enzymes have changed their main substrate preference from DNA to RNA. This interpretation is in agreement with a clustering of DNA and RNA MTases based on structural similarities which led to a similar conclusion [37]. Furthermore, it is strongly supported by the mechanistic data, showing that Dnmt2 methylates RNA with a mechanism which is characteristic for DNA methyltransferases and clearly distinct from RNA methyltransferases [38]. It is interesting to note that bacterial Dnmt2 homologues could be identified so far only in *Geobacter* species, suggesting that they could have been obtained by horizontal gene transfer from eukaryotes.

### Was Dnmt2 the ancestor of Dnmt1 and Dnmt3?

One problem in the hypothetical evolutionary scenario, in which the Dnmt2 is the ancestor of Dnmt1 and Dnmt3 is that all three enzyme were most likely already present in LECA. Moreover, our phylogenetic analysis shows that Dnmt2 enzymes are separated from the Dnmt1 group by numerous branches of prokaryotic DNA MTases, which does not support the model that Dnmt2 is the ancestor of Dnmt1. The situation is less clear for Dnmt3 enzymes. Our phylogenetic analysis shows that among the eukaryotic Dnmt enzymes, Dnmt2 and Dnmt3 share highest sequence similarity, which would support the proposal that they are more closely related. Based on this, it would be of interest to experimentally investigate if Dnmt3 enzyme may methylate RNA, and if yes, if they do so better than other DNA MTases. However, after inclusion of the bacterial enzymes, groups of bacterial enzyme were introduced at different places in the Dnmt2 and Dnmt3 branches in many of the alternative phylogenetic trees. Also Dnmt2 and Dnmt3 enzymes do not cluster in the CLANS analysis. These both points suggest that they have an independent origin.

### Is there an alternative scenario that could be proposed?

Given the very wide distribution of DNA-(cytosine C5)-MTases in different bacteria, it is very likely that prokaryotic DNA MTases predated eukaryotic enzymes. In the phylogenetic tree, the Dnmt2/Dnmt3 group is separated from the Dnmt1 group by several branches of prokaryotic DNA MTases. This result suggests an independent origin of Dnmt1 and Dnmt3 enzymes, which both could have been derived from different bacterial DNA-(cytosine C5)-MTases (Fig. 7), which is in agreement with similar conclusions reached in a recent independent study [39]. Whether Dnmt2 and Dnmt3 are directly related and derived from one common or two different bacterial precursors cannot be decided at present although the second alternative is more likely based on the topologies of alternative trees and the CLANS analyses. In this evolutionary scenario, the Dnmt2 precursor was a DNA-(cytosine C5)-MTase likely part of a bacterial RM system. In LECA this function was lost, because eukaryotes do not possess RM system and the enzyme adopted strong activity for tRNA methylation.

### Conclusions

The ancestor of Dnmt2 was a DNA methyltransferase that changed its substrate to tRNA. There is no phylogenetic evidence that Dnmt2 was the precursor of eukaryotic Dnmt1. Most likely, the eukaryotic Dnmt2, Dnmt1 and Dnmt3 families of methyltransferases had an independent origin in the prokaryotic DNA methyltransferase sequence space and all were derived from MTases of RM systems.

## Methods

### Collection of DNA MTases used for this analysis

The sequences of the DNA methyltransferase proteins belonging to the eukaryotic organisms were retrieved from NCBI non-redundant (nr) database. A collection of bacterial and archeal DNA-(cytosine C5)-MTase was retrieved from REBASE database [25] (http://rebase.neb.com/rebase/rebase.seqs.html). A collection of eukaryotic DNA methyltransferases belonging to different groups were used to retrieve additional sequences from nr (non-redundant) NCBI database using PSI-BLAST algorithm available on the NCBI website. For the searches, gapped blast algorithm was used with default parameters. All the sequences with similarity significance threshold<10^-4^ were collected and duplicates were removed as well as sequences shorter than 150 amino acids. The remaining sequences were clustered using CLANS [Frickey, 2004) and clusters of different MTase groups were extracted. The extracted sequence groups were aligned using ClustalW (using default settings). Afterwards incomplete sequences (i.e. entries missing conserved motifs, in particular motifs I, IV, VI, VIII and X) were removed as well as the N-terminal non-MTase domains of eukaryotic MTases. Sorted and trimmed eukaryotic and prokaryotic methyltransferases were collected and aligned using ClustalW (Text S1) and clustered using CLANS. The NJ tree generated by ClustalW was used to select representative sequences from each major branch of tree with confirmed methylation activity. DRM homologues were treated separately and were retrieved from NCBI non-redundant database by searching Zmet3 (*Z. mays*) and DRM3 (*A. thaliana*) homologues. After aligning the sequences with ClustalW and selecting only unique and complete sequences (Text S2), the sequence permutation was reverted to allow proper alignment with other DNA MTases.

RNA-(cytosine-C5)-methyltransferases where retrieved using PSI-BLAST from NCBI non-redundant database by querying Ncl1p (*S. cerevisae*) and YebU sequences and retrieving all the sequences with score better than E-value 10^-4^. The resulting sequences were aligned using ClustalW and duplicate, incomplete (missing large parts of the MTase domain, missing motif IV or VI) were removed. 260 sequences retained after sorting.

### Multiple sequence alignment

To identify families of closely related sequences, we have generated multiple sequence alignments using T-COFFEE [40] and PCMA [41]. The alignments were manually edited in BioEdit [42]. Guide trees were generated using MEGA 4 [43] using Neighbor-Joining method (JTT model, uniform rates among sites) and for each major cluster of branches a representative sequence was chosen. An alignment of representative sequences was further manually improved by incorporating secondary structure prediction information, structural information derived from available crystal structures, results of the fold recognition and threading servers (using Genesilico metaserver [44]). The final alignment was further striped from non-informative part (such, that only the parts that are homologous to each other were left). During this step, the target recognition regions and variable regions were removed as well.

### Phylogenetic trees

Phylogenetic trees were constructed using the Maximum-Likelihood method with a local installation of PhyML version 3.0 [45]. The tree construction parameters were varied to assess robustness of the generated phylogenies. JTT [46], WAG [47] and Dayhoff models [48], with bootstrap (50-100), were used and the results compared to each other. The generated phylogenies were also tested for consistency, by generating trees using only parts of the alignment (for example removing each of the motif sequences), removing subfamilies of the MTases and constructing phylogenetic trees. The trees were visualized using the MEGA 4 phylogenetic package [43].

We have tried to root the DNA MTase tree using RNA-(cytosine-C5)-methyltransferases as an outgroup. Representative sequences of RNA-(cytosine-C5)-MTases were first aligned with each other and subsequently aligned with the multiple sequence alignment of DNA methyltransferases. The final MSA was refined using the structural information available for YebU (PDB: 2FRX) and a Trm4 homologue (*M.jannaschii* PDB: 3A4T). Unfortunately, as tested by systematic removal of different methyltransferase groups from the analysis, the RNA-(cytosine-C5)-MTases were always clustering with the longest branch on the phylogenetic tree. This phenomenon, called long branch attraction, is a frequently observed problem in phylogenetic analyses, occurring when the sequences are too dissimilar to each other to allow proper inference of phylogeny [49], [50]. This is also the case for our analysis, as the sequence motifs of RNA-(cytosine-C5)-MTases are very distinct from the DNA methyltransferases. Therefore, it was not possible to use the RNA-(cytosine-C5)-MTases to reliably root the DNA MTase/Dnmt2 phylogenetic tree.

### CLANS clustering of DNA-(cytosine-C5)-MTases

The CLANS software [26] was used to generate the all versus all pairwise comparison of the collected sequences and was further used to cluster these sequences based on the pairwise similarity BLASTP score. In the program only scores with a P-value<10^-4^ were used and the clustering process was allowed for more than 4000 cycles to reach completion. After the clustering was completed the separate clusters of all eukaryotic DNA-(cytosine-C5)-MTases were identified, colored and labeled.

## Supporting Information

Text S1 **all_noDRM_raw_alignment.txt.** FASTA file containing the sequences of the catalytic domains of DNA methyltransferases (except the DRM homologues) and Dnmt2 proteins aligned by ClustalW.
(TXT)

Text S2 **DRM_raw_alignment.txt.** FASTA file containing the sequences of the DRM DNA methyltransferases aligned by ClustalW.
(TXT)
